# Incidence of constrained condylar and hinged knee implants and mid- to long-term survivorship: a register-based study from the Nordic Arthroplasty Register Association (NARA)

**DOI:** 10.2340/17453674.2025.42999

**Published:** 2025-02-06

**Authors:** Jake VON HINTZE, Ville PONKILAINEN, Annette W-DAHL, Nils P HAILER, Ove FURNES, Anne M FENSTAD, Mona BADAWY, Alma B PEDERSEN, Martin LINDBERG-LARSEN, Mika J NIEMELÄINEN, Keijo MÄKELÄ, Antti ESKELINEN

**Affiliations:** 1 Coxa Hospital for Joint Replacement and Faculty of Medicine and Health Technologies, Tampere University, Tampere, Finland; 2 Faculty of Medicine and Health Technology, University of Tampere and Tampere University Hospital, Tampere, Finland; 3 The Finnish Arthroplasty Register, National Institute for Health and Welfare, Helsinki, Finland; Department of Orthopaedics and Traumatology, Turku University Hospital, and University of Turku, Turku, Finland; 4 Swedish Arthroplasty Register, and Department of Clinical Sciences Lund, Orthopedics, Lund University, Sweden; 5 Department of Surgical Sciences/Orthopedics & Hand Surgery, Uppsala University Hospital, Uppsala, Sweden; 6 Norwegian Arthroplasty Register, Department of Orthopaedic Surgery, Haukeland University Hospital, Bergen, Norway; 7 Department of Clinical Medicine, University of Bergen, Bergen, Norway; 8 Coastal Hospital in Hagevik, Department of Orthopedic Surgery, Haukeland University Hospital, Bergen, Norway; 9 Danish Knee Arthroplasty Registry, Department of Clinical Epidemiology, Aarhus University Hospital, Denmark; 10 Danish Knee Arthroplasty Registry, Department of Orthopaedic Surgery and Traumatology, Odense University Hospital, Denmark

## Abstract

**Background and purpose:**

In complex primary total knee arthroplasty (TKA), constrained condylar knee (CCK) or rotating hinge knee (RHK) designs may be required to provide stability or address bony deficiencies. We analyzed trends in incidence of these designs in primary TKA and evaluated the mid- to long-term survivorship of CCK and RHK in 4 Nordic countries.

**Methods:**

From 2000 to 2017, 5,134 CCK and 2,515 RHK primary TKAs were identified from the NARA database. Kaplan–Meier (K–M) survival and flexible parametric survival model (FPSM) analyses were performed to estimate revision risk, expressed as hazard ratio (HR) with 95% confidence intervals (CI), with minimally stabilized (MS) TKA acting as the control group (n = 456,137).

**Results:**

The incidence of CCK and RHK implants increased significantly in Finland, while it was moderate in Denmark, Norway, and Sweden. With revision for any reason as the endpoint the 15-year K–M cumulative revision risk for RHK was 13.6% (CI 10.4–16.7) and for CCK it was 11.3% (CI 9.1–13.5). Compared with MS TKA, the hazard ratio for revision was 2.1 (CI 1.8–2.3) for CCK and 2.5 (CI 2.1–2.8) for RHK. Periprosthetic joint infection (PJI) was the most common reason for revision, accounting for 44% of CCK and 47% of RHK cases. After excluding revisions for PJI, the hazard ratio remained high for both designs, at 1.5 (CI 1.3–1.7) for CCK and 1.6 (CI 1.3–2.0) for RHK compared with MS.

**Conclusion:**

The incidence of CCK and RHK increased during the study period. Both designs showed consistent 15-year revision risks of 11–14%, with no major differences between them. The higher revision risk compared with MS TKAs may reflect the complexity of the surgeries.

Most primary total knee arthroplasties (TKA) are performed using cruciate retaining (CR) or posterior stabilizing (PS) components with cemented fixation [1-4]. In complex cases with severe deformities, bony or ligament deficiencies, or issues with knee stability, constrained condylar knee (CCK) or rotating hinge knee (RHK) implant designs are used [5]. During the past decade, a steady increase in the use of CCK and RHK implants in primary TKA has been reported in Norway, the Netherlands, and in National Joint Registry data for England, Wales, Northern Ireland, and the Isle of Man (NJR) [2,6-8].

Prosthetic joint infection (PJI) is reported to be the main reason for revision of CCK and RHK implants in the mid- to long term [2,3,6,9-13]. In recent register studies, survivorship of hinged knee implants in primary TKA has been reported to be around 83% at 10-year follow-up with all primary diagnoses [2,3). In contrast, survival rates for the most used implant models have exceeded 90% at 10-year follow-up [2,3,9,14]. When the survival rates of RHK and CCK implants in primary TKA with only primary osteoarthritis or non-oncological indications at 10-year follow-up are examined, the differences are not substantial [3,9]. However, earlier studies have not compared with a primary minimally stabilized (MS) TKA. This would be of importance to evaluate how the decision to increase degree of constraint perioperatively from an MS implant to a CCK or RHK implant affects the longevity of the TKA in the long term.

The aims of our study were (1) to analyze trends in the incidence of CCK and RHK implants in the 4 participating Nordic countries; and (2) to evaluate the 5-year to 15-year survivorship of the most used CCK and RHK implants compared with primary MS TKAs using data from the Nordic Arthroplasty Register Association (NARA).

## Methods

### Study design and setting

In this observational cohort study, data from NARA were used. NARA compiles data from the Danish Knee Arthroplasty Registry (DKAR), the Norwegian Arthroplasty Register (NAR), the Swedish Arthroplasty Register (SAR), and the Finnish Arthroplasty Register (FAR) [15-18].

The Nordic registries have excellent national coverage and a high degree of completeness for primary knee arthroplasties. The recent completeness of primary knee arthroplasties in the SAR is reported to be 96%, in the DKR 97%, in the NAR 98%, and in the FAR 99%. For revision knee arthroplasties, the corresponding figures are 85% for the SAR, 95% for the DKR, 93% for the NAR, and 92% for the FAR [15-18]. The completeness has steadily increased or remained consistent over the years, despite slight annual variations [16,17]. The combined NARA dataset includes only those variables that all countries register in a uniform way.

### Population

All primary CCK and RHK implants that had been implanted for any reason during the study period were included for incidence analysis. We excluded from the outcome analysis all implant models with fewer than 100 implantations to minimize the learning curve effect, as well as tumor implants. CCK and RHK implants were classified by their femoral component. As there was no information on the level of constraint of the PE inserts, the CCK cohort may have also included knees with a CCK femoral component combined with a PS polyethylene insert.

Cumulative revision rates were assessed and compared separately by model and together by the fixation type of the prosthesis. Revision, defined as the exchange, removal, or addition of 1 or more prosthetic components due to any cause, encompassing such procedures as amputation and arthrodesis, was treated as the endpoint in the survival analysis. In the analyses, the standard MS primary TKA acted as the control group (n = 456,137). MS TKA was defined as an implant that had a flat or dished tibial polyethylene insert regardless of congruency, i.e., both fixed- and mobile-bearing non-PS knees, including CR TKAs with ultra-congruent polyethylene inserts.

### Statistics

Incidence rate was reported as cases per 100,000 person-years. To calculate the incidence rates, the total annual population was obtained from the national official statistics websites of each participating country (www.dst.dk, www.ssb.no, www.scb.se, www.stat.fi). The population was obtained from the last day of each year, except for Denmark, where the population was obtained from the first day of the year. The total annual incidences of RHK and CCK arthroplasties among the 4 Nordic countries were then compared.

Patient and group statistics were described as numbers with percentages, as median with range, or as mean with standard deviation (SD). Kaplan–Meier (K–M) analysis was performed to assess the cumulative revision rates with 95% confidence intervals (CI). Curves were cut off when less than 10% of the total number of knees were at risk. K–M cumulative revision risks were presented separately at 5-year and 10-year time points and by prosthesis type at 10-year and 15-year time points with 95% CI. Competing risk analysis was performed considering revision and death as competing events.

**Figure 1:**
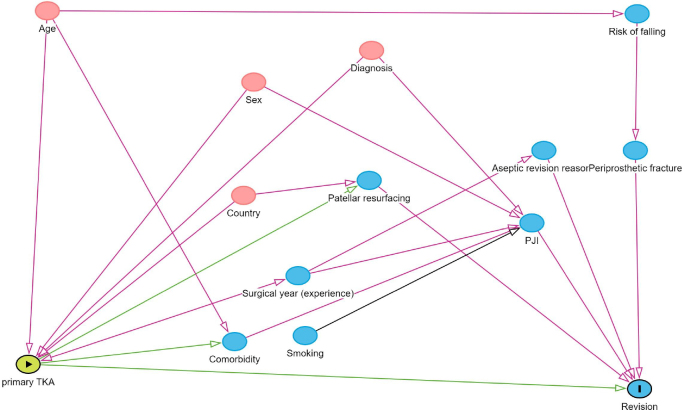
Directed acyclic graph illustrating the determination of adjusted covariates.

Survival analysis was first conducted with multivariable Cox regression models, and proportional hazards assumption was evaluated by checking the correlation of Schoenfeld residuals with time. Remarkable non-proportionality was found that was not solvable with Cox regression using time-dependent coefficients. Therefore, we decided to use a flexible parametric survival model (FPSM) which is not affected by the proportional hazard assumption bias. The FPSM estimates for revision were adjusted for sex, age, country, diagnosis, and year. Year was categorized into the years 2000 to 2009 and 2010 to 2017. The adjusted covariates were chosen based on directed acyclic graphs (Figure 1, see Appendix). The results of the FPSM were reported as hazard ratios (HR) with 95% CI and can be interpreted as in Cox regression models. Exponential distribution was used in the model. The distribution was selected by comparing the distributions with the survival curves and selecting the most similar pattern. Survival analysis was conducted separately for revisions due to any cause and revisions for only aseptic reasons.

Statistical analyses were performed using R 4.0.2 (R Core Team, R Foundation for Statistical Computing, Vienna, Austria).

### Data privacy, ethics, funding, and disclosures

Since the formation of the NARA collaboration, all participating Nordic registries have used individual-based registration of operations and patients. Selection and transformation of the respective datasets and the de-identification of patients, which included the deletion of national civil registration numbers, are performed within each national register. The pseudo-anonymized data was then merged into a common dataset.

The data was treated with full confidentiality in accordance with the rules of the participating countries. The authors of the present study had access only to the common dataset. Data sharing is not possible.

Ethical approval for the study was obtained through the ethical approval process of each national registry. Permission numbers from each country were as follows: the Danish Data Protection Agency (1-16-02-54-17), Denmark, the National Institute of Health and Welfare (Dnro THL/1743/.5.05.00/2014), Finland, the Norwegian Data Inspectorate (ref 24.1.2017: 16/01622-3/CDG), Norway, and the Ethics Board of Lund University (LU20-02), Sweden. The authors have the following potential conflicts of interest to declare. AWD: lecture fees from DePuy; NPH: declares grants/contracts from the Swedish Research Council (VR 2019-00436; VR 2021-00980), Stiftelsen Promobilia, Skobranschens utvecklingsfond, and Waldemar Link; payment/honoraria for lectures, presentations, or educational events from DePuy Johnson & Johnson, Waldemar Link, Zimmer Biomet, and Heraeus Medical; OF: lecture fees from Heraeus Medical & Ortomedic; AE: lecture fees from Heraeus Medical, DePuy Synthes; ABP, AMF, MB, JvH, KM, MLL, MN, and VP: no conflicts of interest. Complete disclosure of interest forms according to ICMJE are available on the article page, doi: 10.2340/17453674.2025.42999

## Results

**Figure 2:**
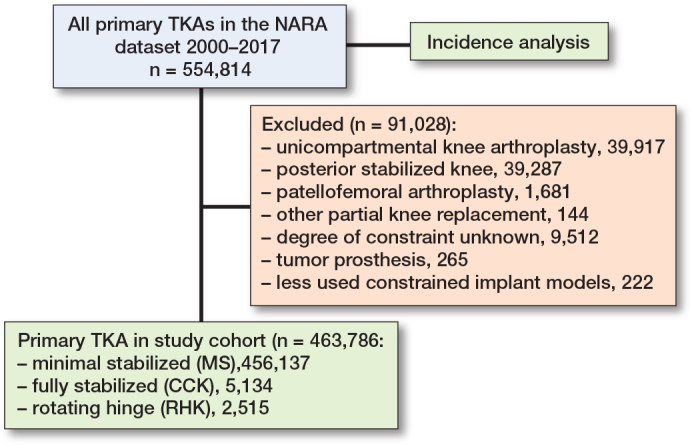
Flowchart of the cohorts. TKA = total knee arthroplasty; NARA = Nordic Arthroplasty Register Association; MS = minimally stabilized knee; CCK = constrained condylar knee; RHK = rotating hinge knee.

554,817 primary knee arthroplasties operated on from January 1, 2000 through December 31, 2017 were identified in the NARA database (Figure 2). A total of 5,134 CCK and 2,515 RHK TKAs were included in the final study cohort. The CCK implant models included in the analyses were NexGen LCCK (Zimmer Biomet, Warsaw, IN, USA), Triathlon TS (Stryker Howmedica Osteonics, Mahwah, NJ, USA), PFC Sigma TC3 (DePuy Synthes, Warsaw, IN, USA), Duracon Total Stabilizer (Stryker Howmedica Osteonics, Mahwah, NJ, USA), AGC Revision (Biomet, Warsaw, IN, USA), and Vanguard Constrained (Zimmer Biomet, Warsaw, IN, USA). The included RHK implant models were NexGen RHK (Zimmer Biomet, Warsaw, IN, USA), Endo-Model (Waldemar LINK GmbH and Co, Hamburg, Germany), MRH (Stryker Howmedica Osteonics, Mahwah, NJ, USA), and S-ROM Noiles (DePuy Synthes, Warsaw, IN, USA).

**Figure 3:**
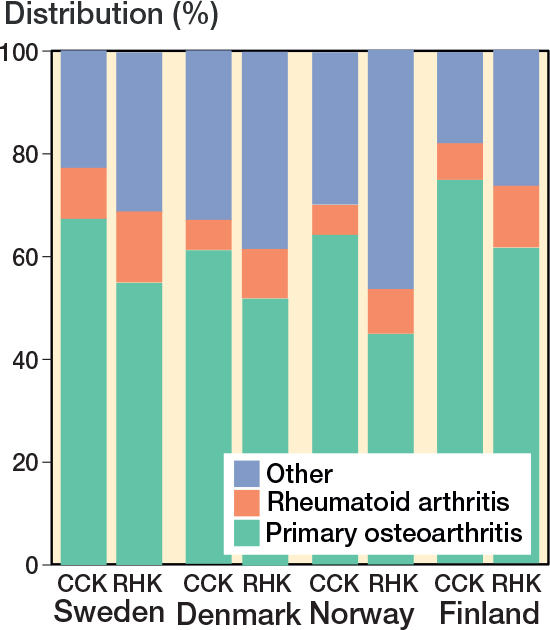
Differences in the diagnosis proportion for CCK and RHK prostheses among the Nordic countries from 2000 to 2017. For Abbreviations, see Figure 2.

**Figure 4:**
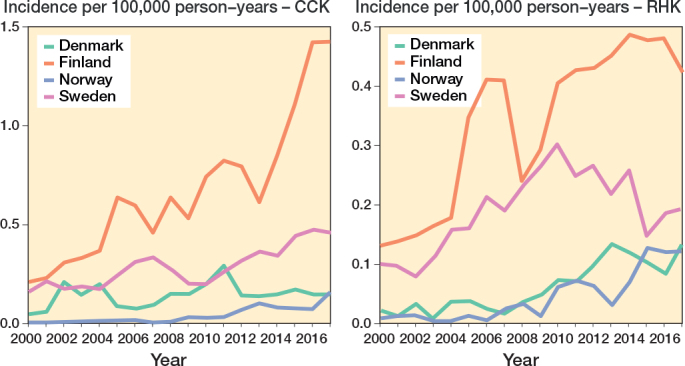
Age- and sex-adjusted incidence rates for CCK and RHK implants in 4 Nordic countries between 2000 and 2017. For Abbreviations, see Figure 2.

The demographics of the study cohorts showed that there were intergroup differences in the distribution of indications and age groups, as well as intercountry differences in the use of different implants and patellar buttons (Table 1, Figure 3, see Appendix). Of the 4 Nordic countries, Finland had the highest incidence of both CCK and RHK procedures over the study period (Figure 4). In 2017, the incidence rates of CCK procedures were 1.4 per 100,000 person-years in Finland, 0.5 in Sweden, 0.2 in Norway, and 0.2 in Denmark. In the same year, the corresponding rates for RHK procedures were 0.4 in Finland, 0.2 in Sweden, and 0.1 in Norway and Denmark.

**Table 1:** Demographics of the RHK, CCK, and MS study cohorts

Factor	RHK	CCK	MS
No. of arthroplasties	2,515	5,134	456,137
Median age (range)	72 (14–98)	70 (18–95)	70 (9–101)
Females, %	78	72	62
Follow-up, years median (range)	4.2 (0–18)	4.3 (0–18)	6.0 (0–18)
Age category, n (%)			
< 55	283 (11)	540 (10)	33,322 (7.3)
55–64	435 (17)	1,057 (21)	112,345 (25)
65–74	737 (29)	1,788 (35)	177,464 (39)
≥ 75	1,060 (42)	1,749 (34)	133,006 (29)
Country, n (%)			
Denmark	223 (8.9)	616 (12)	81,478 (18)
Norway	167 (6.6)	172 (3.4)	63,039 (14)
Sweden	739 (29)	1,270 (25)	173,301 (38)
Finland	1,386 (55)	3,076 (60)	138,319 (30)
Indications, n (%)			
Primary osteoarthritis	1,448 (58)	3,643 (71)	418,004 (92)
Rheumatoid arthritis	303 (12)	391 (7.6)	14,784 (3.2)
Other	764 (30)	1,100 (21)	23,349 (5.1)
Patellar button, %	201	24	22
Denmark	65	60	79
Norway	19	20	3.9
Sweden	2.8	6.9	6.1
Finland	23	24	18
No. of revisions	178	323	17,005

For Abbreviations see Figure 2.

**Figure 5:**
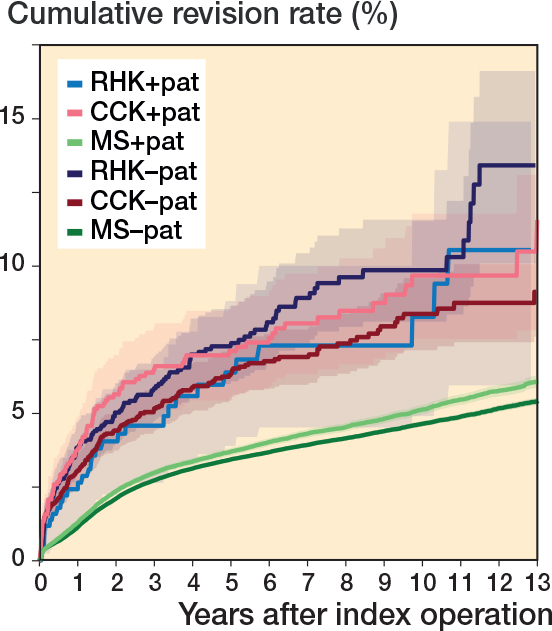
Kaplan–Meier curves for cumulative revision rate (with 95% confidence interval) with patellar versus without patellar component for the MS, CCK, and RHK groups. For Abbreviations, see Figure 2.

**Figure 6:**
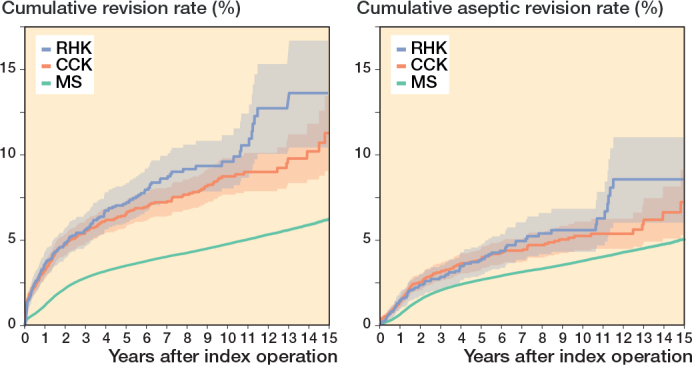
Kaplan–Meier curves for cumulative revision rate (with 95% confidence interval) for the MS, CCK, and RHK groups with revision for any reason and for only aseptic reasons as the endpoint. For Abbreviations, see Figure 2.

The 10-year K–M cumulative revision risk was 9.6% for RHK implants, 8.7% for CCK, and 4.8% for MS, with revision for any reason as the endpoint. At 15 years, risks were 13.6%, 11.3%, and 6.2%, respectively. Only small differences were found between the CCK and RHK groups regarding patellar resurfacing (Table 2, Figure 5, see Appendix). Excluding PJI-related revisions, the 10-year risks were 5.6% for RHK, 5.2% for CCK, and 3.8% for MS. At 15 years, they were 8.5%, 7.2%, and 5.0%. The RHK and CCK revision risks remained comparable over 10 years (Figure 6).

**Table 2:** Different fixation types with Kaplan–Meier cumulative revision risks with 96% confidence intervals (CI), flexible parametric survival mode (FPSM) analysis with hazard ratio (HR), and follow-up years with interquartile range (IQR) in the NARA database during the period 2000–2017

Type	n	10-year follow-up		15/13-year follow-up ^a^		FPSM HR (CI)	Follow-up median (IQR)
		Number at risk	Revision risk (CI)	Number at risk	Revision risk (CI)		
MS	456,137	106,264	4.8 (4.7–4.9)	20,747	6.2 (6.1–6.4)	1	6.0 (2.8–9.7)
CCK	5,134	813	8.7 (7.7–9.8)	132	11.3 (9.1–13.5)	2.1 (1.8–2.3)	4.3 (1.7–8.0)
RHK	2,515	323	9.6 (8.0–11.2)	37	13.6 (10.4–16.7)	2.5 (2.1–2.8)	4.2 (1.8–7.5)
MS–pat.	354,272	78,378	4.7 (4.6–4.8)	33,450	5.4 (5.3–5.6)	1	5.8 (2.8–9.4)
MS+pat.	101,865	27,886	5.1 (5.0–5.3)	13,257	6.1 (5.9–6.3)	0.8 (0.8–0.8)	6.6 (3.1–10.4)
CCK–pat.	3,916	559	8.4 (7.2–9.6)	239	9.2 (7.6–10.7)	2.0 (1.7–2.3)	3.9 (1.6–7.5)
CCK+pat.	1,218	254	9.7 (7.6–11.8)	82	11.6 (8.2–14.9)	1.8 (1.5–2.2)	5.6 (2.1–9.2)
RHK–pat.	1,998	237	9.9 (8.1–11.6)	70	14.7 (10.6–18.6)	2.6 (2.2–3.0)	4.2 (1.9–7.4)
RHK+pat.	517	86	8.3 (4.9–11.6)	28	10.6 (6.0–15.0)	1.6 (1.1–2.3)	3.9 (1.7–8.0)

a 15-year data for main categories (MS, CCK, and RHK: see Legend Figure 2 for Abbreviations) and 13-year data for use of patellar component.

–pat. = implant without patellar component.

+pat. = implant with patellar component.

Compared with MS TKA, the HR was 2.1 (CI 1.8–2.3) for CCK implants and 2.5 (CI 2.1–2.8) for RHK implants in FPSM analysis. After excluding revisions for PJI, the HR was 1.5 (CI 1.3–1.7) for CCK implants and 1.6 (CI 1.3–2.0) for RHK implants.

**Figure 7:**
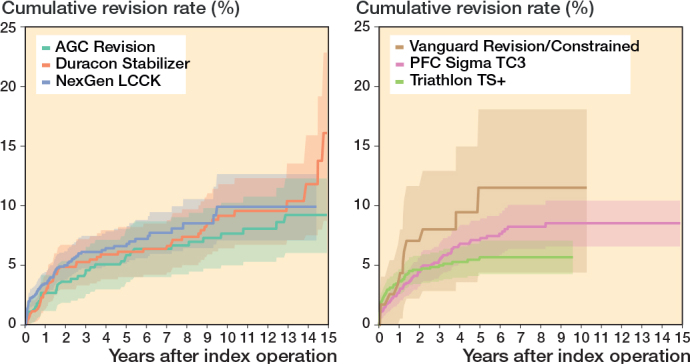
Kaplan–Meier curves for cumulative revision rate (with 95% confidence interval) for the CCK implants with revision for any reason as the endpoint.

**Figure 8:**
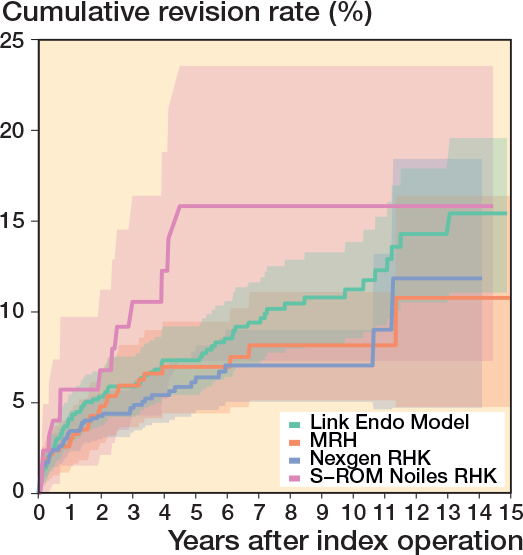
Kaplan–Meier curves for cumulative revision rate (with 95% confidence interval) for the rotating hinge knee implants with revision for any reason as the endpoint.

**Figure 9:**
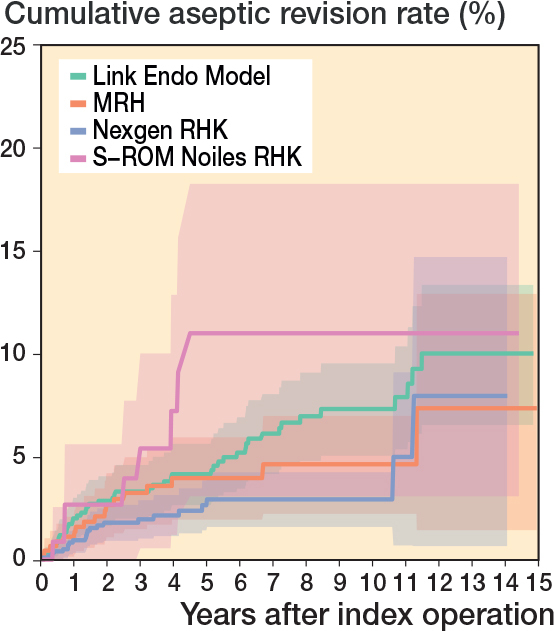
Kaplan–Meier curves for cumulative revision rate (with 95% confidence interval) excluding prosthetic joint infections for the RHK models.

**Figure 10:**
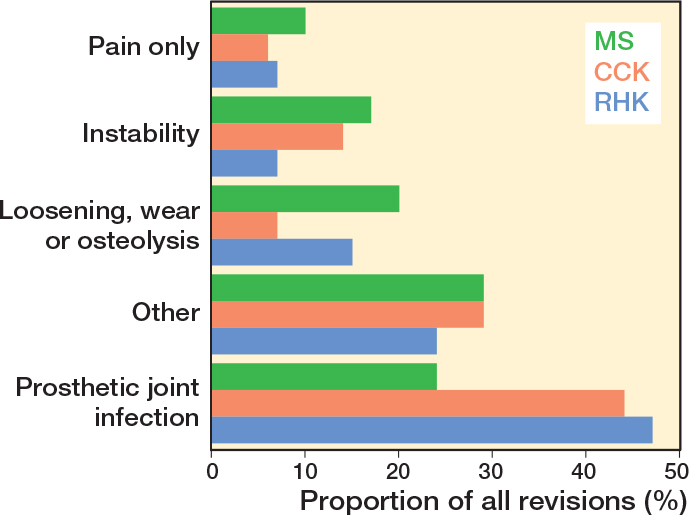
Distribution of reasons for revision in the MS, CCK, and RHK groups. For Abbreviations, see Figure 2.

**Figure 11:**
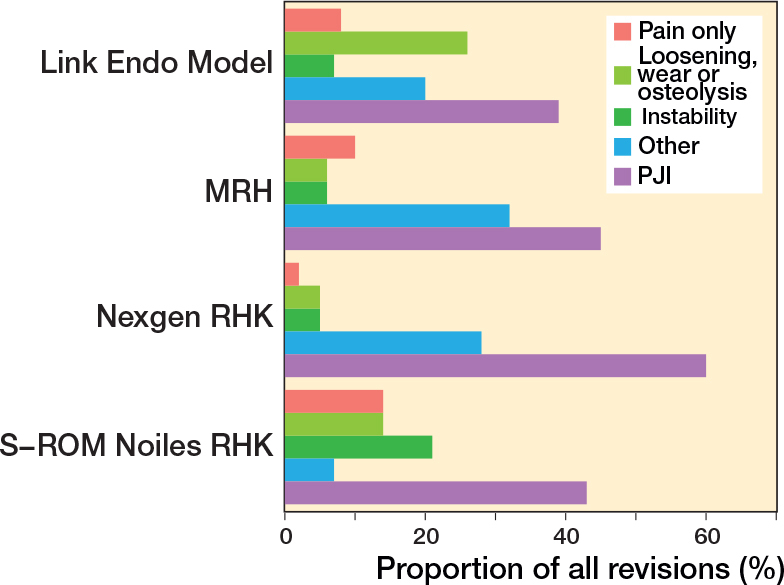
Distribution of reason for revision by RHK models. PJI = periprosthetic joint infection.

**Figure 12:**
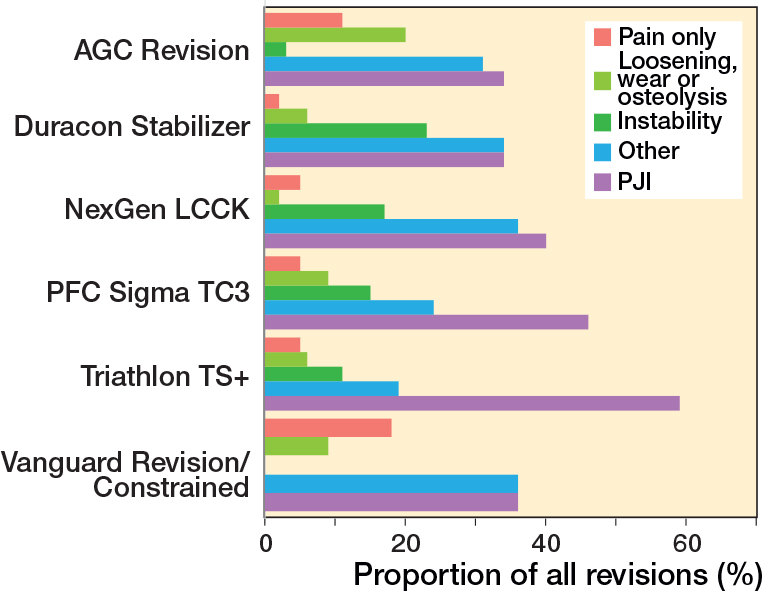
Distribution of reason for revision by CCK models. PJI = periprosthetic joint infection.

In the CCK group, there were no marked differences in 10-year survival risks between the implant designs (Figure 7, Table 3). In the RHK group, only S-ROM Noiles evinced more than a 10% revision risk at 5 years, even when PJIs were excluded (Figure 8, Table 3, Figure9, see Appendix). At 10 years, the revision risks of the NexGen RHK and MRH designs did not exceed 10%. PJI was the main reason for revision in both the CCK and the RHK groups (Figure 10). Furthermore, PJI was also the dominant reason for revision for all of the CCK and RHK designs (Figure 11 and 12, see Appendix). Estimated HRs of PJI from FPSM showed that age or year of surgery had no effect on the revision risk in relation to PJI. On the other hand, both CCK and RHK implants were associated with an increased risk compared with MS implants, as well as with male sex (Table 4).

**Table 3:** Most used CCK and RHK implants with Kaplan–Meier cumulative revision risks, FPSM analysis, and follow-up years in the NARA database during the period 2000–2017

Type	n	5-year follow-up		10-year follow-up		FPSM HR (CI)	Follow-up median (IQR)
		Number at risk	Revisionrisk (CI)	Number at risk	Revision risk (CI)		
CCK group							
NexGen LCCK	1,479	483	6.6 (5.1–8.1)	105	9.9 (7.1–12.7)	1.4 (0.9–2.2)	2.9 (1.2–6.1)
Triathlon TS+	1,344	445	5.7 (4.3–7.1)			1.2 (0.7–2.0)	3.3 (1.2–5.8)
PFC Sigma TC3	1,224	562	7.1 (5.5–8.8)	204	8.6 (6.6–10.5)	1.1 (0.7–1.7)	4.5 (1.8–8.5)
Duracon Stabilizer	520	410	6.2 (4.0–8.2)	252	9.2 (6.4–11.8)	1.5 (0.9–2.3)	9.8 (6.1–12.3)
AGC Revision	449	364	5.9 (3.6–8.1)	251	7.7 (5.0–10.3)	1	11 (6.3–14)
Vanguard Constrained	118	43	12 (4.4–18)			1.7 (0.8–3.4)	4.0 (2.6–6.6)
RHK group							
Nexgen RHK	1,087	345	6.4 (4.6–8.2)	54	7.0 (5.0–9.0)	0.6 (0.4–0.9)	3.3 (1.6–6.0)
Link Endo Model	811	482	7.3 (5.4–9.2)	186	11 (8.6–14)	1	6.3 (3.2–9.7)
MRH	484	215	7.0 (4.4–9.5)	72	8.2 (5.1–11.1)	0.8 (0.5–1.3)	4.3 (1.8–7.4)
S-ROM Noiles	133	37	16 (7.3–24)	11	16 (7.3–24)	1.5 (0.8–2.7)	2.9 (1.1–5.5)

For Abbreviations, see Table 2.

**Table 4:** Estimated hazard ratios (HR) of prosthetic joint infection from flexible parametric survival model (FPSM) analysis

Factor Category	Reference	FPSM HR (CI)
Primary diagnosis		
Primary OA	Other	0.7 (0.6–0.8)
Primary RA	Other	1.3 (1.1–1.6)
Primary diagnosis for CCK		
Primary OA	Other	0.7 (0.5–1.1)
Primary RA	Other	0.4 (0.2–0.9)
Primary diagnosis for RHK		
Primary OA	Other	1.1 (0.7–1.9)
Primary RA	Other	0.8 (0.4–1.6)
Age		
All		1.0 (0.99–1.0)
CCK		1.0 (0.98–1.0)
RHK		1.0 (0.97–1.0)
Sex		
All – Men	Women	1.9 (1.8–2.0)
CCK – Men	Women	1.9 (1.3–2.7)
RHK – Men	Women	2.3 (1.4–3.6)
Sweden		
CCK	MS	5.0 (3.7–6.6)
RHK	MS	5.4 (3.8–7.7)
Denmark		
CCK	MS	3.1 (2.0–4.8)
RHK	MS	5.0 (2.6–9.6)
Norway		
CCK	MS	2.1 (0.7–6.6)
RHK	MS	6.7 (3.5–12.7)
Finland		
CCK	MS	3.2 (2.4–4.1)
RHK	MS	3.2 (2.2–4.7)
Year		
All		1.1 (1.1–1.2)
CCK		1.0 (1.0–1.1)
RHK		1.0 (0.9–1.0)

OA = osteoarthritis.

RA = rheumatoid arthritis.

For other Abbreviations, see Figure 2.

**Figure 13:**
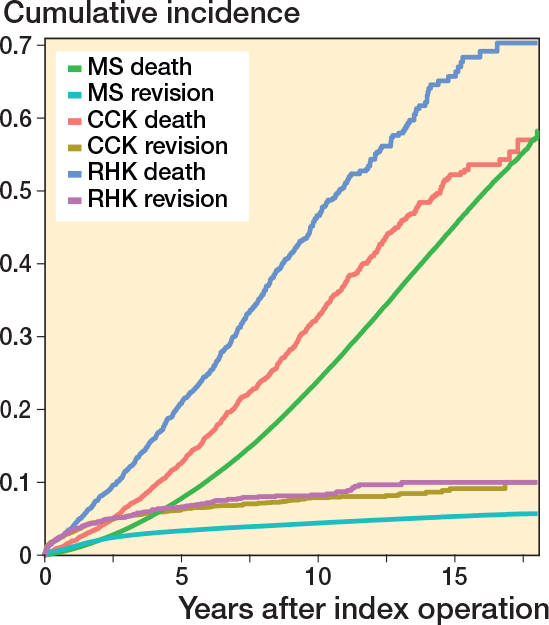
Competing risk analysis for death and revision surgery as competing events over time (in years). For Abbreviations, see Figure 2.

Competing risk analysis for probability of revision (MS 5.4%, CCK 9.2%, RHK 10.1% at 15 years) and death (MS 45.6%, CCK 52.4%, RHK 66.0% at 15 years) as competing events is shown in Figure 13, see Appendix.

## Discussion

We aim to analyze trends in incidence for CCK and RHK designs in primary TKA and evaluate their mid-term survivorship. We found substantial differences in the incidence of CCK and RHK arthroplasties over time among 4 Nordic countries. Revision rates between CCK and RHK during the 15 years of follow-up were, however, comparable.

Finland had the highest incidence and largest increase in CCK and RHK procedures over the 18-year period. In Sweden, RHK incidence remained stable from 2004 to 2017, while it increased steadily in Denmark and Norway. CCK incidence in Denmark rose slightly after peaking in 2011. The rise in RHK and CCK procedures in Finland from 2004 to 2007 was likely due to long waiting times for surgery: as the deformity of the end-stage osteoarthritic knee worsens over time, patients are more likely to need a constrained knee implant if the waiting lists are very long. Moreover, the increase in CCK procedures after 2014 may be linked to patients’ new right to choose their healthcare providers: many patients had waited a long time for TKA, and they were now allowed to choose a public hospital from hospital districts other than their own. Patients with deformed osteoarthritic knees suddenly gained access to surgery after prolonged waiting times. Irmola et al. have reported that the increase in incidence of primary TKA decreased somewhat in the Nordic countries between 2000 and 2017 [1]. Deehan et al. (2022) revealed a 66% increase in the use of hinged knee arthroplasties between 2011 and 2017 in the NJR [7]. Further, based on data from the Norwegian Arthroplasty Register, Badawy et al. (2019) previously demonstrated an increased use of hinged and CCK implants in primary TKA between 2005 and 2017 [6]. In 2017, the NJR reported that 0.1% of all primary cemented procedures were hinged and 1.1% were CCKs [2]. Similarly, data from the Dutch Arthroplasty Register (LROI) for the same year revealed that 0.3% of all primary TKA procedures were hinged and 0.3% were CCKs [8].These figures are in line with NARA data. Of all the primary knee arthroplasties recorded in the NARA database in 2017, 0.5% were RHKs and 1.3% CCKs.

We have demonstrated that revision risks were comparable between the CCK and RHK groups during the 15-year follow-up. As expected, survivorship of MS knees was significantly better than that of CCK or RHK knees. This finding possibly derives from the higher complexity of surgeries and from differences in indications between groups. This is supported by the fact that in RHK and CCK groups more patients were operated on for reasons other than primary OA (e.g., secondary OA due to sequelae of knee trauma). Additionally, in the competing risk analysis, the mortality over 15 years of follow-up is higher in the RHK (66%) and CCK (52%) groups compared with the MS group (46%). However, the difference in revision risks between MS and CCK/RHK knees became less evident when PJIs were excluded. We also defined the reasons for revision for each CCK and RHK design in primary TKA, with PJI being the main reason for revision for each design. There were no major differences in revision risks or reasons for revision between the different implant designs.

The K–M analysis of 10-year revision risks showed comparable outcomes between the RHK (9.6%) and the CCK groups (8.7%). Moreover, the comparable mechanical performance was emphasized when 10-year revision risks were examined after excluding revisions for PJI. Subsequently, the divergence in cumulative revision risks becomes clearer between the 2 groups after 10 years. The 15-year K–M cumulative revision risks were 13.6% and 11.3% for the RHK and CCK groups, respectively. However, reports from the NJR and the Australian arthroplasty registry (AOANJRR) show more divergence between these 2 groups at an earlier stage [2,3]. According to the NJR, the cumulative revision risk for cemented CCK implants was 2.7% at 5 years postoperatively. In contrast, pre-assembled/hinged/linked fixation type implants had a 5.9% revision risk at 5 years postoperatively when both rotating and linked implants were included [2]. The AOANJRR reported the cumulative revision risk for fully stabilized CCK implants to be 6.2% at 5 years and 8.1% at 10 years postoperatively. Furthermore, the cumulative revision risk for hinged implants was 11.3% at 5 years and 17.6% at 10 years postoperatively [3]. When compared with the findings of these registries, we found that the RHK group had a more acceptable and comparable revision risk in mid-term follow-up. However, selection bias complicates the interpretation of these results, as our analysis contained only the most used implant designs that had been implanted more than 100 times, whereas the AOANJRR included all types of hinge implants (as well as tumor prostheses) in their results.

In a registry-based study, Castagnini et al. (2022) reported 10-year postoperative survival risks of 93.4% and 91.9% for CCK and RHK, respectively [9]. These results are comparable with our data. In contrast, 10-year postoperative survival risks have been reported for primary CCK of between 79% and 90% and for RHK of between 75% and 87% [6,13,19–21].

Our findings regarding the 10-year K–M cumulative revision rate among patients undergoing patellar resurfacing in both the RHK and CCK groups do not align with other studies. For example, the NJR has reported improved outcomes for cemented hinged implants without patellar resurfacing versus with patellar resurfacing. Moreover, the NJR has also reported improved outcomes for cemented CCK with patellar resurfacing than without patellar resurfacing [2].

In our study, the most common reason for revision in both the CCK and the RHK groups was PJI. This is consistent with previous published findings in the literature [6,10,13,22-24]. Unfortunately, a more detailed analysis of the different aseptic causes for revision could not be performed due to the limited variables of the different reasons for revision in our data. Several studies have shown, however, that when septic revision cases are excluded, survival rates have improved for both CCK and RHK type implants when revision for aseptic reasons is considered as the only endpoint of follow-up [6,12,13,25]. In contrast, Martin et al. (2016) reported poor long-term survival risks for RHK type implants (74.6% at 10 years and 40.3% at 20 years). In their study, the patients were relatively young in the RHK group and tumors were included in the study, which may have affected the outcomes [13]. It has previously been reported that most patients receiving a constrained implant in a primary setting are typically older females [2,6,9,23]. Moreover, when compared with unconstrained type implants, indication is more often rheumatoid arthritis or secondary OA than primary OA [3,9,13].

### Limitations

First, lower completeness of revision TKA (than primary TKA) is a factor that may have affected survival rates. However, it is unlikely that the completeness of revision TKA is differentially dependent on the registration of CCK or RHK implants. Second, preoperative patient-reported outcome measures (PROMs) and ASA scores are not available in the NARA database and could not be included as confounders in our Cox regression analyses [26].Third, a comparison of different aseptic failure types was not possible due to the limited variables for different reasons. Moreover, some implant designs may have had stem and augment modifications that we were unable to ascertain. Additionally, no clear consensus exists in the literature on stem usage for primary TKA [9,27-30]. Fourth, additionally, according to the results of the competing risk analysis, the FPSM results may underestimate the true revision risk, especially in the RHK group, as mortality is quite high during the follow-up. Fifth, all patients who have received a CCK or an RHK implant may not be directly comparable to those who have been operated on with an MS TKA, as suggested by the differences in indications for surgery.

### Strengths

The major strength of our study is the NARA dataset, which covers a large number of less frequently used constrained and hinged knee arthroplasties in a primary setting. Therefore, we were able to compare the different implant designs and determine the incidences for these designs separately among the 4 Nordic countries. Our study included data on the CCK and RHK models, which were lacking in the previous literature, enabling the comparison of the different implants. The results of this study may provide clinicians with tools for implant selection, as well as for shared decision-making with patients.

### Conclusions

We found variable trends in incidences of CCK and RHK implants during the study period, with Finland having the highest incidence. Revision risks for the CCK and RHK implants were 8.7% and 9.6% at 10-year follow-up and 11.3% and 13.6% at 15-year follow-up, respectively, which were higher than for MS implants. There were comparable aseptic revision rates between the RHK and CCK during 15-year follow-up in a primary TKA setting but PJI still remains the most common reason for revision for these implants.

## Notes

Handling co-editor: Per Henrik Randsborg

*Acta* thanks Peter Bobak, Marc J Nieuwenhuijse, and other anonymous reviewers for help with peer review of this manuscript.
