# Incidence of constrained condylar and hinged knee implants and mid- to long-term survivorship: a register-based study from the Nordic Arthroplasty Register Association (NARA)

**DOI:** 10.2340/17453674.2025.44996

**Published:** 2025-11-10

**Authors:** Jake VON HINTZE, Ville PONKILAINEN, Annette W-DAHL, Nils P HAILER, Ove FURNES, Anne M FENSTAD, Mona BADAWY, Alma B PEDERSEN, Martin LINDBERG-LARSEN, Mika J NIEMELÄINEN, Keijo MÄKELÄ, Antti ESKELINEN

**Affiliations:** 1 Coxa Hospital for Joint Replacement and Faculty of Medicine and Health Technologies, Tampere University, Tampere, Finland; 2 Faculty of Medicine and Health Technology, University of Tampere and Tampere University Hospital, Tampere, Finland; 3 The Finnish Arthroplasty Register, National Institute for Health and Welfare, Helsinki, Finland; Department of Orthopaedics and Traumatology, Turku University Hospital, and University of Turku, Turku, Finland; 4 Swedish Arthroplasty Register, and Department of Clinical Sciences Lund, Orthopedics, Lund University, Sweden; 5 Department of Surgical Sciences/Orthopedics & Hand Surgery, Uppsala University Hospital, Uppsala, Sweden; 6 Norwegian Arthroplasty Register, Department of Orthopaedic Surgery, Haukeland University Hospital, Bergen, Norway; 7 Department of Clinical Medicine, University of Bergen, Bergen, Norway; 8 Coastal Hospital in Hagevik, Department of Orthopedic Surgery, Haukeland University Hospital, Bergen, Norway; 9 Danish Knee Arthroplasty Registry, Department of Clinical Epidemiology, Aarhus University Hospital, Denmark; 10 Danish Knee Arthroplasty Registry, Department of Orthopaedic Surgery and Traumatology, Odense University Hospital, Denmark

We noted an error in the age- and sex-adjusted incidence rates, which have now been corrected in the Results section and in Figure 4. We are sorry for this mistake.

On behalf of the authors

Jake von Hintze

## Original version

The demographics of the study cohorts showed that there were intergroup differences in the distribution of indications and age groups, as well as intercountry differences in the use of different implants and patellar buttons (Table 1, Figure 3, see Appendix). Of the 4 Nordic countries, Finland had the highest incidence of both CCK and RHK procedures over the study period (Figure 4). In 2017, the incidence rates of CCK procedures were 1.4 per 100,000 person-years in Finland, 0.5 in Sweden, 0.2 in Norway, and 0.2 in Denmark. In the same year, the corresponding rates for RHK procedures were 0.4 in Finland, 0.2 in Sweden, and 0.1 in Norway and Denmark.

## Corrected version

The demographics of the study cohorts showed that there were intergroup differences in the distribution of indications and age groups, as well as intercountry differences in the use of different implants and patellar buttons (Table 1, Figure 3, see Appendix). Of the 4 Nordic countries, Finland had the highest incidence of both CCK and RHK procedures over the study period (Figure 4). In 2017, the incidence rates of CCK procedures were 6.9 per 100,000 person-years in Finland, 1.2 in Sweden, 0.8 in Norway, and 0.7 in Denmark. In the same year, the corresponding rates for RHK procedures were 2.0 in Finland, 0.6 in Norway and Denmark, and 0.5 in Sweden.

## Original version

**Figure 4:**
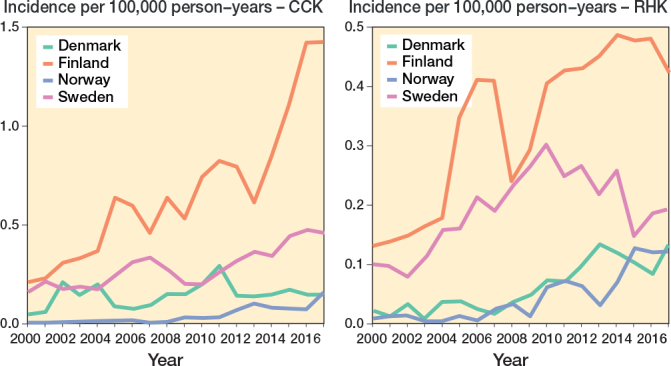
Age- and sex-adjusted incidence rates for CCK and RHK implants in 4 Nordic countries between 2000 and 2017. For Abbreviations, see Figure 2.

## Corrected version

**Figure 4:**
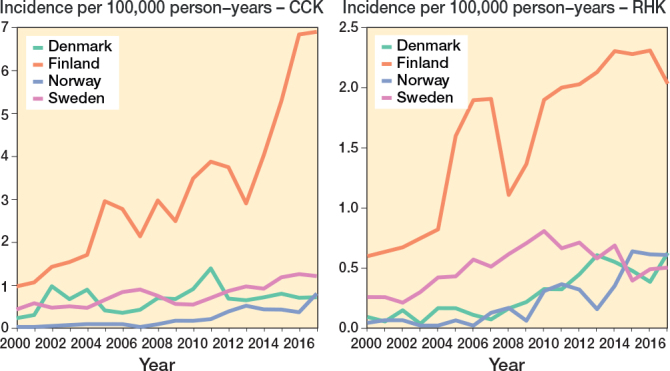
Age- and sex-adjusted incidence rates for CCK and RHK implants in 4 Nordic countries between 2000 and 2017. For Abbreviations, see Figure 2.

Figure 2. Flowchart of the cohorts. TKA = total knee arthroplasty; NARA = Nordic Arthroplasty Register Association; MS = minimally stabilized knee; CCK = constrained condylar knee; RHK = rotating hinge knee.

